# Treatment Patterns in Essential Tremor: A Retrospective Analysis

**DOI:** 10.5334/tohm.682

**Published:** 2022-03-23

**Authors:** Chintan Shah, George R. Jackson, Aliya I. Sarwar, Pitchaiah Mandava, Fariha Jamal

**Affiliations:** 1 Parkinson’s Disease Center and Movement Disorders Clinic, Department of Neurology, Baylor College of Medicine, Houston, Texas, US; 2 Parkinson’s Disease Research, Education, and Clinical Center, Michael E. DeBakey Veterans Affairs Medical Center, and Department of Neurology, Baylor College of Medicine, Houston, Texas, US; 3 Center for Alzheimer’s and Neurodegenerative Diseases, Baylor College of Medicine, Houston, TX, US; 4 Department of Neurology, Baylor College of Medicine, Houston, Texas, US; 5 Neurology Care line, Michael E. DeBakey VA Medical Center, Houston, TX, US

**Keywords:** essential tremor, treatment, medication, clinical, botulinum toxin, deep brain stimulation

## Abstract

**Background:**

Although first line therapies for essential tremor have been identified from small clinical trials, responses are variable. We conducted a survey of tremor management in a large sample of ET cases.

**Methods:**

The Movement Disorders Clinical Case Registry within a US Veterans Health Administration medical center was used to identify 1468 patients with ET.

**Results:**

Of 1468 charts reviewed, 1074 (73.19%) met criteria for ET with characterization of temporal course and treatment; 291/1074 subjects (27.1%) did not receive any treatment. Almost half (500/1074; 46.6%) of the patients received monotherapy, 196/1074 (18.2%) two, 66/1074 (6.1%) three, and 21/1074 (2.0%) four or more medications. Of all prescriptions, primidone was the most used (546/1172; 46.6%), followed by propranolol (419; 35.8%), topiramate (122; 10.4%) and gabapentin (35; 3.0%). Medication response was available for a total of 1030 prescriptions, of which 138 (13.4%) were discontinued due to side effects; 180 (17.5%) prescriptions were ineffective. Furthermore, 52/1074 patients (4.8%) were treated with botulinum toxin injections and 41/1074 (3.8%) underwent deep brain stimulation surgery.

**Discussion:**

Our data suggest that more widespread recognition of limitations underlying conventional approaches, as well as increased referrals for nonpharmacological therapies, may be necessary to achieve improved outcomes in ET populations.

## Introduction

Essential tremor (ET) is the most common movement disorder in the elderly, with an estimated prevalence of 4.6% above 65 years of age and 0.9% in the general population [1,2]. The definition of ET has changed over time, and diagnostic criteria vary significantly among clinicians. In 2018, the International Parkinson and Movement Disorder Society Task Force on Tremor published an updated consensus statement on the classification of tremors [3]. They defined ET as an isolated tremor syndrome of bilateral upper limb action tremor of at least 3 years duration, with or without tremor in other locations (e.g., head, voice, or lower limbs). ET is not associated with other neurological signs, such as dystonia, ataxia, or parkinsonism. ET progression is insidious, and symptoms may remain mild for long periods and not require any treatment; however, over time, tremor progression may be severe enough to cause functional limitations, necessitating treatment. The decision to treat ET is influenced by several factors, including severity, functional limitations, co-morbidities, other medications, and patient preference. Although propranolol was the first treatment used for tremor in 1971, the list of available pharmacotherapies has not grown much over time. The International Parkinson and Movement Disorder Society task force conducted an evidence-based review of treatments for ET in 2019 and concluded that although the disease is chronic and progressive with disabling tremor experienced by most patients, treatment options are very limited. Current pharmacologic and non-pharmacologic therapies are focused only on the symptomatic treatment of upper limb tremor. After decades of research, propranolol and primidone remain the most efficacious agents, with high doses of topiramate also showing clinical efficacy [4]. The response to pharmacological therapies remains highly variable with adverse effects limiting efficacy [5].

According to a large patient-centered survey of ET, 16.1% of the respondents reported that their current treatment for tremor was insufficient [6]. Another data-based survey of 678 patients concluded that propranolol and primidone were considered effective by only 40% of patients [7]. Most treatment data derive from trials of individual medications; however, surveys examining overall treatment patterns in ET patients are limited.

We reviewed treatment patterns in a large cohort of veterans diagnosed with ET in a Veterans Affairs Medical Center movement disorders clinic. Our aim was to answer certain questions including percentage of patients who chose not to take medication due to mild tremor, number of patients who did not continue the treatment due to lack of efficacy versus development of adverse effects, individual medication responses, combination of medications, and percentage of patients opting for chemo denervation or deep brain stimulation.

## Methods

### Study design

The Veterans Health Administration is one of the oldest health systems in the country using electronic medical records. Every encounter requires entry of International Disease Classification (ICD)-9/10 codes. Several applications have been employed to establish databases for clinical, research and administrative purposes. The Movement Disorders Clinical Case Registry (MD-CCR) is an application that works within a specific US Veterans Health Administration medical center’s electronic medical record to query and export information. The MD-CCR in Houston was queried for all patients with a diagnosis of essential tremor according to the International Disease Classification (ICD)-9/10 codes 333.1 (essential and other specified forms of tremor) and/or G25.0 (essential tremor) seen in a movement disorder specialty center’s outpatient clinic (the Parkinson’s Disease Research, Education, and Clinical Center; PADRECC) between September 1, 2001 and March 31, 2018. This query generated a cohort of 1468 patients with the diagnosis of ET, based on ICD codes. Each patient’s record was reviewed by a senior neurology resident (C.S.) and/or a movement disorders physician (F.J. or G.J) to verify the diagnosis and to obtain information on treatment provided. This retrospective study was approved by the Baylor College of Medicine (BCM) Institutional Review Board and the VA Research and Development Committee.

### Population studied

Of the 1468 patients yielded by the registry based on ICD codes, we were able to verify a diagnosis of ET along with detailed characterization of age of onset and treatment patterns, in 1074 patients based on the criteria defined by the consensus statement of the International Parkinson and Movement Disorder Society. The remaining 394 patients were excluded due to a diagnosis of Parkinson’s disease, dystonic tremor, enhanced physiologic tremor, drug-induced tremor, cerebellar tremor, functional tremor, or Huntington’s disease. Data were obtained and analyzed retrospectively from each patient’s record and entered into a database that included fields for gender, age at tremor onset, age at diagnosis, number of medications trialed, medications, and medication response. Medication response was patient driven and rated as no response, mild, moderate, or significant. Reasons for discontinuation were extracted for each medication and tabulated under “no response” and/or “development of adverse effects”. We did not track medications used primarily for Parkinson’s disease or tremor-predominant Parkinson’s disease, such as levodopa, trihexyphenidyl, or dopamine agonists, as it was not possible to distinguish antiparkinsonian from anti-tremor effects in those patients that met diagnostic criteria for ET in addition to one of these diagnoses. Data regarding use of botulinum toxin injections (muscles injected) and deep brain stimulation (DBS) surgery (choice of target) were also extracted.

### Statistical analysis

We performed a descriptive analysis of categorical variables such as number of medications trialed, type of medication prescribed, muscles injected with botulinum toxin, and choice of DBS target reported as number of cases and percentage. Treatment response for each medication was graded as no response, mild, moderate, significant, or unable to tolerate. Assessment of the response was based on subjective perception and feedback provided by the subjects. No changes in tremor severity and ability to perform activities of daily living was marked as no response whereas mild improvement in severity but no changes in functional abilities requiring further medication adjustment was labeled as mild response. Subjects reporting improvement in both severity and functional abilities but still requiring some adaptive strategies was classified as moderate response. Improvement in tremor severity and functional abilities with no use of adaptive strategies was marked as significant response. Continuous variables such as age at tremor onset, age at diagnosis, age at first botulinum toxin injection, and number of botulinum toxin treatment visits were presented as mean ± standard deviation. Graphs were constructed using Graphpad Prism (San Diego, CA). In some cases, the Kolmogorov-Smirnov two sample test was used in Graphpad Prism to assess differences between groups.

## Results

**Figure 1:**
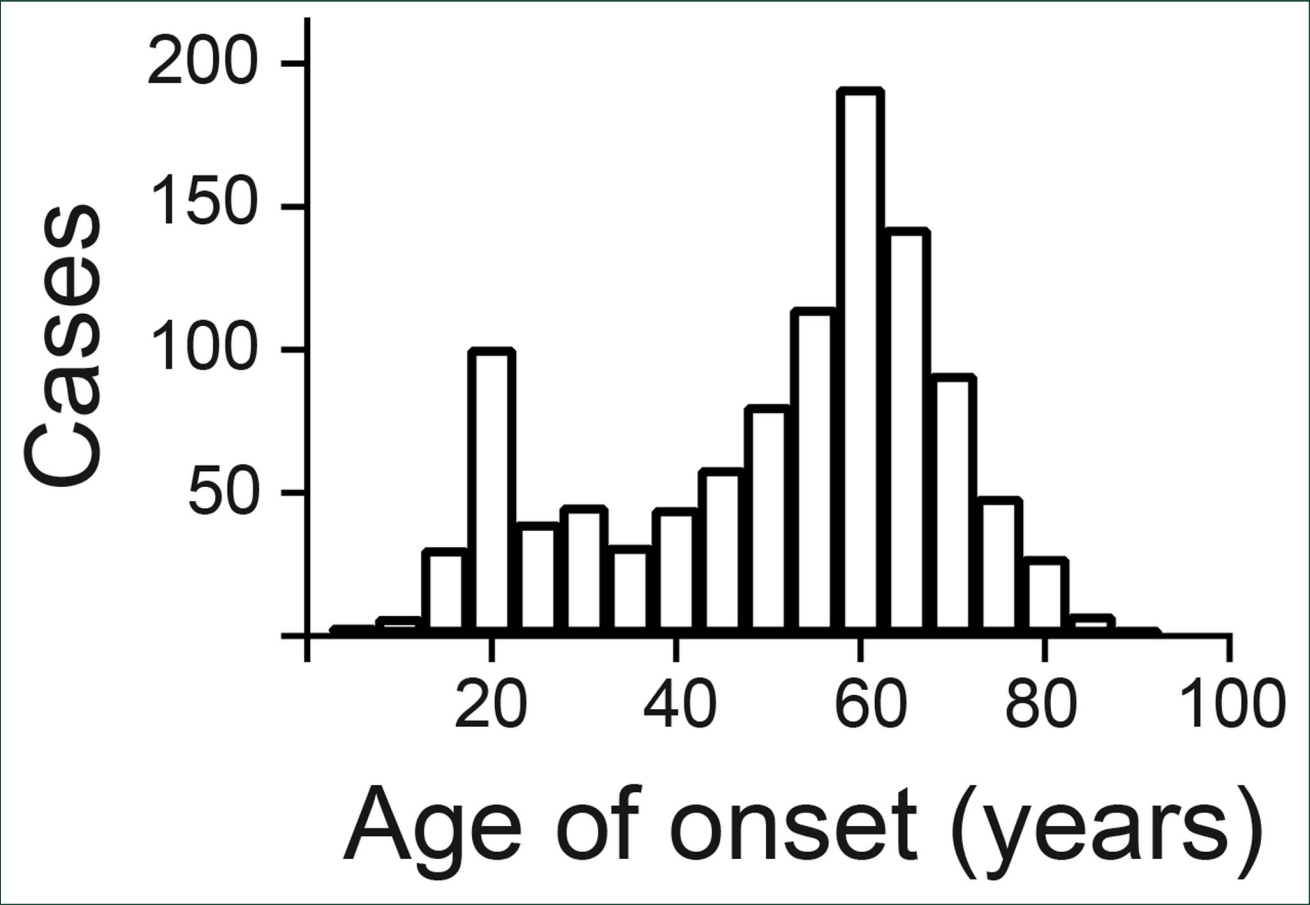
Distribution of Age at onset of Essential Tremor.

The MD-CCR query generated a set of 1468 patients based on ICD 9/10 codes between Sept. 1, 2001 (the Houston PADRECC was first established in 2001) and March 31, 2018. Each of these patients had been evaluated in person by a movement disorder specialist. After review of records and exclusion of patients that did not meet diagnostic criteria for ET, we analyzed a set of 1074 patients, each of whom had data regarding age at onset and treatment. The overall age of onset data did not meet criteria for a normal pattern of distribution (Anderson-Darling, D’Agostino and Pearson, Shapiro-Wilk, and Kolmogorov-Smirnov tests) but appeared to be bimodal (***Figure 1***). Of the 1074 subjects, 1030 (95.9%) were male and 44 (4.1%) female. Age of tremor onset was 51.10 ± 18.43 for men and 47.18 ± 16.87 for women (mean ± SD), *p* < 0.001, Kolmogorov-Smirnov test). We did not find significant differences in age of onset for race or ethnicity. Subjects were between 22 and 94 years of age at initial evaluation (64.0 +/– 10.3; mean +/– SD). Just over a quarter of the subjects (291/1074; 27.1%) did not receive any treatment (***Table 1***). 500 (46.5%) patients were treated with one, 196 (18.2%) with two, 66 (6.1%) with three, and 21 (2.0%) were treated with four or more medications (***Table 1***).

**Table 1:** Overview of medication use in 1074 ET patients.


Confirmed cases of ET with treatment data	**1074**

No treatment	291 (27.1%)

One medication	500 (46.5%)

Two medications	196 (18.2%)

Three medications	66 (6.1%)

Four or more medications	21 (2.0 %)

Total number of prescriptions	**1172**

Primidone	546 (46.6%)

Propranolol	419 (35.8%)

Topiramate	122 (10.4%)

Gabapentin	35 (3.0%)

Benzodiazepines (clonazepam + diazepam)	24 (2.0%)

Metoprolol	20 (1.7%)

Atenolol	3 (0.2%)

Nadolol	3 (0.2%)

Total number of Rx w/medication response	**1030**

Side effects leading to discontinuation	138

No response	180


Of a total of 1172 prescriptions, 546 (46.6%) were for primidone, 419 (35.8%) for propranolol, 122 (10.4%) for topiramate, and 35 (3.0%) for gabapentin (***Table 1***). Medication response was available for a total of 1030 prescriptions, of which 138 (13.4%) were discontinued due to side effects; 180 (17.5%) prescriptions had no effect. Approximately one fourth (25/96; 26.0%) of topiramate prescriptions were associated with side effects leading to discontinuation; gabapentin (4/31; 12.9%), primidone (64/490; 13.1%), and propranolol (41/366; 11.2%) were somewhat better tolerated. Almost half (10/22; 45.5%) of benzodiazepine prescriptions were ineffective, with topiramate (27/96; 28.1%), gabapentin; (10/31; 32.3%), and other beta-blockers (5/25; 20.0%, excluding propranolol) showing slightly more favorable responses.

**Figure 2:**
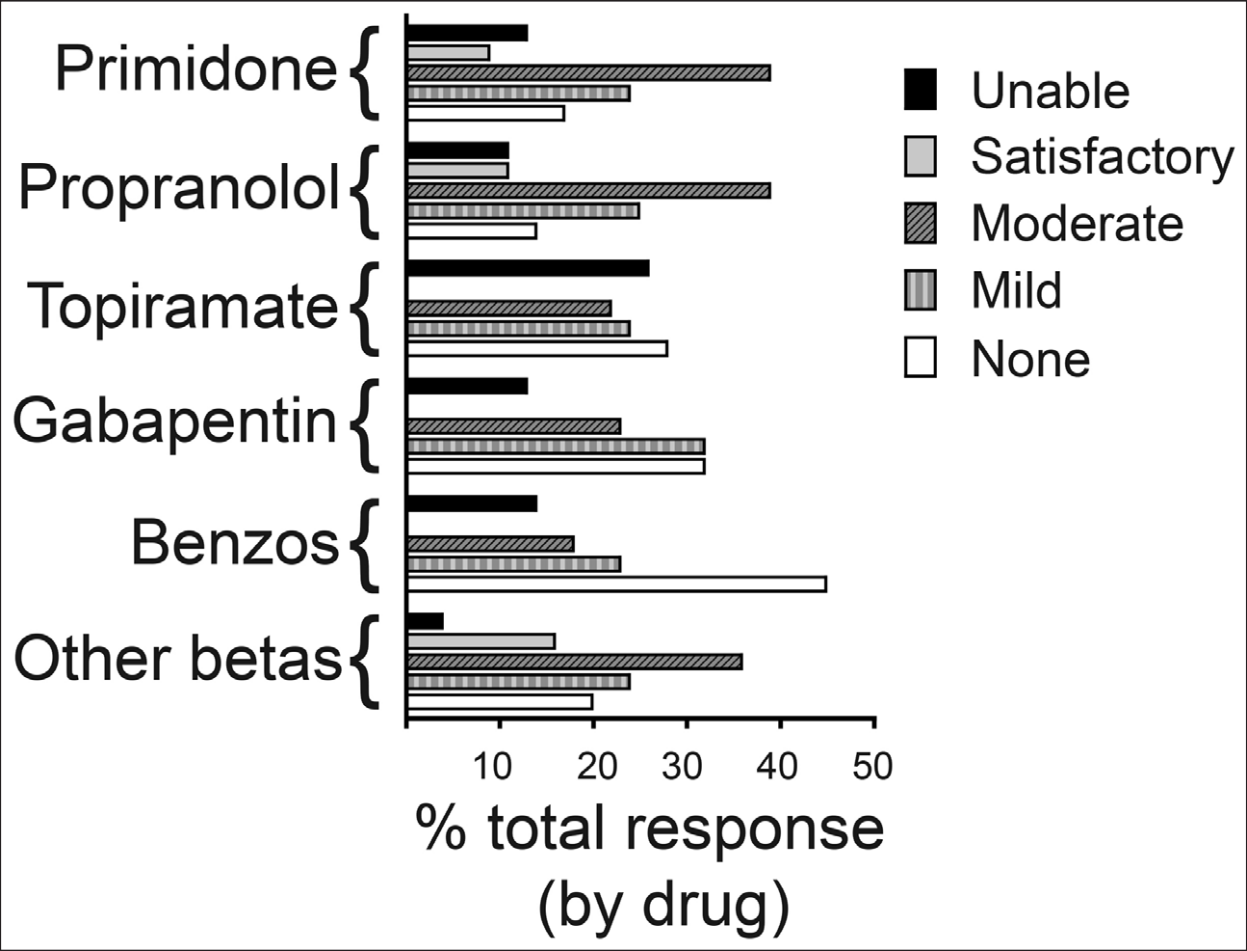
Tremor response based on individual treatment.

Tremor responses to individual drugs are summarized in ***Figure 2***. Tremor improvement with primidone was as follows: none (77/490; 15.7%), mild (116; 23.7%), moderate (190; 38.8%), and significant (43; 8.8%). A similar pattern was noted with propranolol: no response (51/366; 13.9%), mild (91; 24.97%), moderate (144; 39.3%), and significant (39; 10.7%). For topiramate, there was either no response (27/96; 28.1%), mild (23; 24.0%), or moderate (21; 21.9%); for gabapentin, no response (10/31; 32.3%), mild (10; 32.3%), or moderate (7; 22.6%); for benzodiazepines, no response (10/22; 45.5%), mild (5; 22.7%), or moderate (4; 18.2%); and for beta-blockers other than propranolol, no response (5/25; 20.0%), mild (6; 24.0%), moderate (9; 36.0%), and significant (4; 16.0%). Although 856/1030 (83.1%) of the prescriptions with response data were for primidone or propranolol (first line agents), 318/1030 (30.9%) of these were associated with lack of efficacy or side effects leading to discontinuation.

Fifty-two patients (4.8%) were treated with botulinum toxin injections for medically refractory upper limb tremor. The mean age at first injection was 67.8 ± 11.4 years and the mean number of treatment visits was 5.2 ± 4.0 (***Table 2***). Medical therapy was continued for 47 (90.4%) patients at the time of first injection visit. The number of patients receiving their first botulinum toxin injection doubled from 26.9% to 53.8% over a 11-year period between 2007 and 2018 (***Table 2***).

**Table 2:** Non-pharmacological treatments provided to ET patients.


Number of patients receiving botulinum toxin (n = 1074)	**52 (4.8%)**

2001–2006	10 (19.2%)

2007–2012	14 (26.9%)

2013–2018	28 (53.8%)

Mean age at first injection	67.8 ± 11.4

Mean number of treatment visits	5.2

Median number of treatments	4

Medications + Botulinum toxin	47 (90.4%)

Unilateral limb injections	27 (51.9%)

Bilateral limbs injections	25 (48.1%)

Muscle Distribution Pattern (n = 52)	

Forearm flexors	49 (94.2%)

Supinators	23 (44.2%)

Forearm extensors	19 (36.5%)

Pronator teres	10 (19.2%)

Number of patients receiving DBS (n = 1074)	41 (3.8%)

Unilateral VIM (n = 41)	4 (9.8%)

Unilateral VIM (n = 41)	37 (90.2%)


Injections were administered in unilateral upper limb for 27 (51.9%) patients and in bilateral upper limbs for 25 (48.1%) patients. Forearm flexors were the site of injection in 49 (94.2%) patients followed by supinators in 23 (44.2%) patients, forearm extensors in 19 (36.5%) patients, and pronator teres in 10 (19.2%) patients (***Table 2***).

Among the 41 (3.8%) patients who underwent deep brain stimulation (DBS) surgery, 37 (90.2%) patients underwent bilateral placement of electrodes at ventral intermedius nucleus (VIM) of the thalamus. Four (9.8%) of the total DBS patients received only unilateral VIM DBS.

## Discussion

Our retrospective analysis of over 1000 patients provides important insight into treatment patterns for essential tremor. Age of onset showed bimodal distribution similar to previously published work. Female patients showed earlier age at onset than male patients, but the total number of female veterans was much smaller than the male veteran cohort, likely reflecting changing demographics of the US armed forces with increased participation by women in recent years. Approximately one quarter of our cohort did not receive treatment due to patient preference and/or non-disabling tremor, like a previous survey reviewing medication usage patterns in ET patients [8]. About a quarter of our ET patients required trials of multiple therapies, with a small proportion of patients having tried four or more medications. The need for multiple medication trials highlights the limited efficacy of pharmacological therapies in general. The number of patients taking multiple medications was much lower in our cohort as compared to the survey conducted by Diaz and Louis (26.3% vs. 73.1%) [8]. This difference may reflect the unique nature of veteran populations, with many suffering mood disorders or chronic obstructive pulmonary disease, thus limiting choices due to contraindications or risk of adverse drug interactions.

Primidone and propranolol, well-known first-line agents for ET, were also the most prescribed medications in our cohort. Primidone was prescribed more often than propranolol likely due to a large proportion of veterans with contraindications to propranolol such as asthma, diabetes, or treatment with other beta-blockers. Tremor response between primidone and propranolol was similar, with about three quarters of prescriptions associated with tremor improvement. However, significant tremor reduction from each medication was identified in only a small proportion of prescriptions. This level of therapeutic response to both agents has been documented previously in smaller studies and is also replicated in our large cohort [9]. Approximately one fourth of primidone and propranolol prescriptions were associated with lack of response or adverse effects that necessitated medication discontinuation. A similar large survey reported medication discontinuation rate as high as 30% among ET patients [10]. In our cohort, primidone was associated with slightly higher rates of intolerable side effects than propranolol. However, the rate of primidone discontinuation in our cohort was not as high as previously reported [8,11,12]. This disparity may reflect differences in starting doses, titration strategies, and level of tolerability in the veteran population. These data emphasize the limited efficacy of primidone and propranolol, despite being first line treatments.

Topiramate is classified as a first line tremor medication and as per recent Movement Disorder Society evidence-based review is considered efficacious for upper limb tremor. However, our study demonstrates poor efficacy, with none of the patients reporting significant tremor reduction. Furthermore, topiramate showed the highest rate of adverse effects compared to other the treatments prescribed in our cohort. Ondo et al. reported that adverse events were treatment limiting in 31.9% patients taking topiramate and 9.5% of placebo patients [13]. These authors observed a 29% improvement in overall tremor scores with topiramate at a mean final dose of 292 mg/day. One explanation for the better improvement noted previously could be related to relatively higher dose of topiramate, which were not noted in our cohort (data not shown). A small proportion of our cohort received second line agents such as gabapentin and benzodiazepines. Tremor response was variable with these agents, with most prescriptions demonstrating lack of efficacy or only mild tremor improvement, as reported in previous studies [14,15]. Interestingly, beta blockers other than propranolol demonstrated tremor response similar to propranolol or primidone, but the total number of prescriptions for other beta blockers was smaller in comparison to first line agents. Prior reports also have demonstrated similar efficacy between propranolol and other selective beta blockers such as metoprolol and atenolol [16,17,18]. Our results indicate that selective beta blockers are reasonable treatment options in patients who cannot receive treatment with non-selective beta blockers such as propranolol or those who have already been prescribed selective beta blockers for other conditions.

With no significant breakthroughs in pharmacotherapy and limitations of current medications, there has been a surge of interest in botulinum toxin in treatment of ET over the last 25 years. Our study spanning 17 years also demonstrates a substantial increase over time in the proportion of patients receiving their first botulinum toxin injection for upper limb tremor. Most of these patients were continued on medications but still preferred to pursue botulinum toxin injections given their unsatisfactory treatment response. Multiple studies have demonstrated efficacy of botulinum toxin in the treatment of limb tremor, with individualized injection strategies demonstrating significant tremor improvement [19,20,21]. In our cohort, veterans also were injected with an individualized approach to upper limb tremor, with forearm flexors being the most common site of injection, followed by supinators. A study using a similar injection strategy, with the majority of limb injections being in the forearm flexors and supinators, demonstrated sustained tremor improvement with low risk of focal hand weakness [22].

Our cohort has the advantage of a large sample size and a vast amount of treatment data available for review from electronic medical records. However, we acknowledge the limitations of our work given its retrospective design. There is limited generalizability, with all of our subjects representing a single movement disorders clinic from a Veterans Affairs medical center with a sizeable male preponderance. Treatment response data based on ethnicity and race was not extracted. Furthermore, the degree of treatment response to each medication was obtained subjectively from a patient’s perceived response instead of objective measures such as validated clinical tremor rating scales. Another limitation is that we did not collect detailed data on adverse effects that led to medication discontinuation. Additionally, given the retrospective design of the study, we were unable to compare individual medication responses to combined medication responses. For example, primidone and propranolol when prescribed together have shown synergistic effect on tremor reduction [23]. Lastly, we excluded patients who had ET early on but later went on to develop Parkinson’s disease (PD). The treatment response to ET medications in the ET → PD group would be of important value.

Although primidone and propranolol can confer reasonable tremor reduction in many ET patients, their use is limited by side effects or pre-existing contraindications, particularly in aging populations with multiple medical problems and polypharmacy. Many of the other medications used for ET studied have demonstrated variable response, highlighting the limitations of current pharmacological therapies. Recent advances in non-pharmacological interventions such as individualized botulinum toxin injections and deep brain stimulation, and MR-guided focused ultrasound have extended tremor benefit in patients with medically refractory tremor. Our data suggest that more widespread recognition of limitations underlying conventional approaches, as well as increased referrals for nonpharmacological therapies, may be necessary to achieve improved outcomes in ET populations.
